# Functional Energetics of CD4^+^-Cellular Immunity in Monoclonal Antibody-Associated Progressive Multifocal Leukoencephalopathy in Autoimmune Disorders

**DOI:** 10.1371/journal.pone.0018506

**Published:** 2011-04-20

**Authors:** Aiden Haghikia, Moritz Perrech, Bartosz Pula, Sabrina Ruhrmann, Anja Potthoff, Norbert H. Brockmeyer, Susan Goelz, Heinz Wiendl, Hans Lindå, Tjalf Ziemssen, Sergio E. Baranzini, Tor-Björn Käll, Dietmar Bengel, Tomas Olsson, Ralf Gold, Andrew Chan

**Affiliations:** 1 Department of Neurology, St. Josef-Hospital, Ruhr-University Bochum, Bochum, Germany; 2 Department of Dermatology and HIV Competence Center, St. Josef-Hospital, Ruhr-University Bochum, Bochum, Germany; 3 Biogen Idec, Cambridge, Massachusetts, United States of America; 4 Department of Neurology, Westfälische Wilhelms-Universität Münster, Münster, Germany; 5 Neurology Unit, Department of Medicine, Karolinska Institute, Danderyd Hospital, Stockholm, Sweden; 6 Department of Neurology, Technical University, Dresden, Germany; 7 Department of Neurology at the University of California San Francisco, San Francisco, California, United States of America; 8 Clinic of Internal Medicine Södersjukhuset, Stockholm, Sweden; 9 Neurologic Clinic, Oberschwabenklinik, Ravensburg, Germany; 10 Department of Neurology, Karolinska Hospital, Stockholm, Sweden; University of California San Francisco, United States of America

## Abstract

**Background:**

Progressive multifocal leukoencephalopathy (PML) is an opportunistic central nervous system- (CNS-) infection that typically occurs in a subset of immunocompromised individuals. An increasing incidence of PML has recently been reported in patients receiving monoclonal antibody (mAb) therapy for the treatment of autoimmune diseases, particularly those treated with natalizumab, efalizumab and rituximab. Intracellular CD4^+^-ATP-concentration (iATP) functionally reflects cellular immunocompetence and inversely correlates with risk of infections during immunosuppressive therapy. We investigated whether iATP may assist in individualized risk stratification for opportunistic infections during mAb-treatment.

**Methodology/Principal Findings:**

iATP in PHA-stimulated, immunoselected CD4^+^-cells was analyzed using an FDA-approved assay. iATP of mAb-associated PML (natalizumab (n = 8), rituximab (n = 2), efalizumab (n = 1)), or other cases of opportunistic CNS-infections (HIV-associated PML (n = 2), spontaneous PML, PML in a psoriasis patient under fumaric acids, natalizumab-associated herpes simplex encephalitis (n = 1 each)) was reduced by 59% (194.5±29 ng/ml, mean±SEM) in comparison to healthy controls (HC, 479.9±19.8 ng/ml, p<0.0001). iATP in 14 of these 16 patients was at or below 3^rd^ percentile of healthy controls, similar to HIV-patients (n = 18). In contrast, CD4^+^-cell numbers were reduced in only 7 of 15 patients, for whom cell counts were available. iATP correlated with mitochondrial transmembrane potential (ΔΨ_m_) (iATP/ΔΨ_m_−correlation:tau = 0.49, p = 0.03). Whereas mean iATP of cross-sectionally analysed natalizumab-treated patients was unaltered (448.7±12 ng/ml, n = 150), iATP was moderately decreased (316.2±26.1 ng/ml, p = 0.04) in patients (n = 7) who had been treated already during the pivotal phase III trials and had received natalizumab for more than 6 years. 2/92 (2%) patients with less than 24 months natalizumab treatment revealed very low iATP at or below the 3^rd^ percentile of HC, whereas 10/58 (17%) of the patients treated for more than 24 months had such low iATP-concentrations.

**Conclusion:**

Our results suggest that bioenergetic parameters such as iATP may assist in risk stratification under mAb-immunotherapy of autoimmune disorders.

## Introduction

PML is a demyelinating, potentially fatal opportunistic infection of the CNS incited by the JC polyomavirus (JCV). Despite a high seroprevalence of anti-JCV-antibodies in healthy adults, estimated to be ∼80%, development of PML in non-immunocompromised individuals is very rare [1]. Conditions that predispose to PML are typically linked to defects of CD4^+^-/CD8^+^-cell-mediated immunity, with HIV infection accounting for about 80% of all new PML-cases [2], [3]. Recently, however, cases of PML have been observed upon immunotherapy with monoclonal antibodies (mAbs), including natalizumab, rituximab and efalizumab, that have been approved for the treatment of multiple sclerosis (MS), Crohn's disease; non-Hodgkin lymphoma, chronic lymphocytic leukaemia, rheumatoid arthritis; and psoriasis, respectively [4]–[7]. Of these mAb immunotherapy-associated PML cases, 85 have been confirmed as of January 7^th^ 2011 to be specifically natalizumab-associated. All cases had received natalizumab in the drug's post-marketing phase during which it was marketed along with a “Black Box” warning that included the risk of PML development (www.fda.org).

While the precise pathogenic mechanism of mAb-associated PML remains elusive, the involvement of CD4^+^- and CD8^+^-lymphopenia is postulated as a risk factor in at least some of these patients [5]. Moreover, rapid reconstitution of CNS-immunosurveillance is predicted to lead to the control of PML [8]. However, the effective resurrection of the immune system after removal of respective mAb with plasma exchange (PLEX) and/or immunoadsorption (IA) is associated with an immune reconstitution inflammatory syndrome (IRIS), that is characterised by an inflammatory brain infiltrate consisting of lymphocytes and multinucleated cells [9], [10].

Additional evidence suggests an important role for CD4^+^-/CD8^+^-cell-mediated immunity in the anti-JCV immune response. Recognition of extracellular, MHC-class II-presented viral antigens by CD4^+^-cells and subsequent activation of cytotoxic CD8^+^-cells appears to be important for the control of JCV-infected cells [11]–[14], and an association of HLA-class I haplotypes and CD8^+^-cellular responses with prognosis of PML has been reported [15], [16].

For a resting T-cell to become an activated immune effector cell it must experience a phenotypic and functional shift that requires an enhanced supply of ATP-generating metabolites to meet the increased bioenergetic demands of the activated cell state [17]. The ability of lymphocytes to import energy-carrying metabolites and to upregulate oxidative phosphorylation appears to be critical in the maintenance of effective immune responses [17].

Here we set out to assess bioenergetic properties as a measure of cellular immunocompetence in PML and other opportunistic CNS-diseases. We used an FDA-approved assay for the detection of cell-mediated immunity in an immunosuppressed population (FDA no. k013169). This assay measures ATP-concentration in CD4^+^-cells (iATP), which correlates with cytokine secretion and T-cell proliferation and thus serves as a measure of T-cell activation [18], [19]. Furthermore, in immunosuppressed renal transplant recipients, low iATP has been found to be associated with the risk of reactivation of BK-virus, another opportunistic human polyomavirus similar to JCV [20]. Our data, obtained using samples from MS patients under mAb therapy and in pathogenetically diverse PML patients as well as HIV patients were also corroborated through a biochemically independent method.

## Results

### Reduced CD4^+^-iATP in PML-patients, in patients with opportunistic CNS-infections and HIV-patients

**Figure 1:**
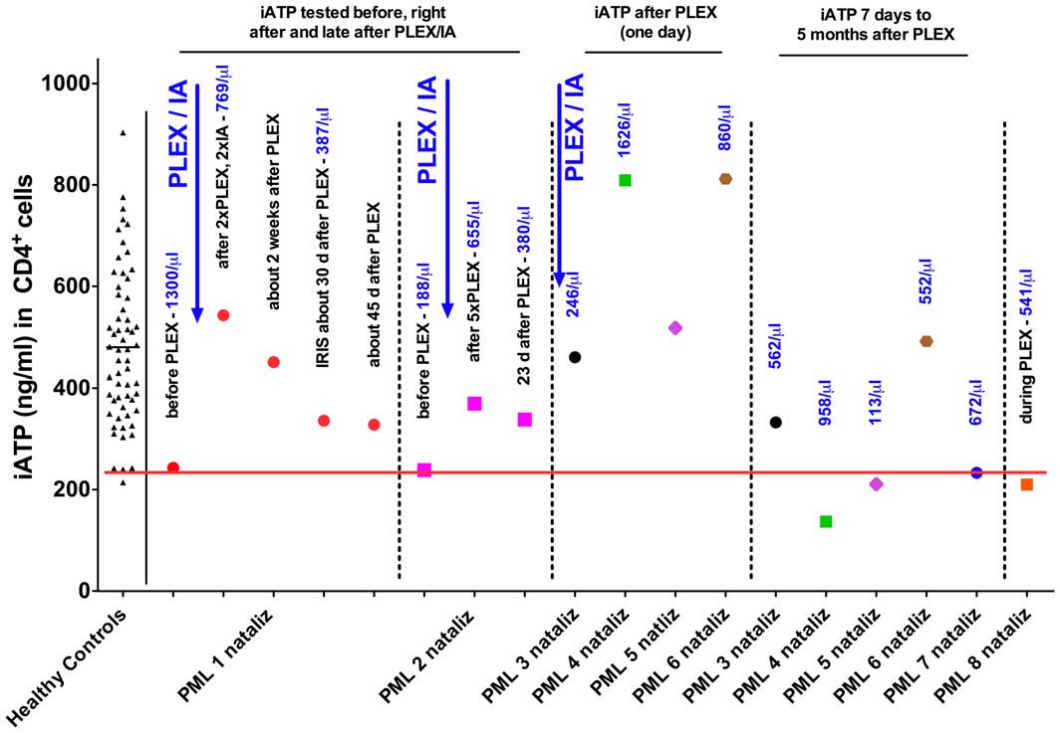
Intracellular CD4^+^-ATP-concentrations in natalizumab-associated PML. MS patients who developed progressive multifocal leukoencephalopathy (PML) under natalizumab-treatment. For two patients (PML nataliz 1 and 2) samples were available at time point of diagnosis (before plasma exchange/immunoadsorption; PLEX/IA), immediately after and at different time points after PLEX/IA as indicated. For PML nataliz 3–8 samples were only available during/after therapy; results are shown immediately after PLEX/IA (PML after PLEX) and at least one week after PLEX/IA (PML 7 days to 5 months after PLEX). Total number of CD4^+^-cells per microliter (µl) and clinical/therapeutic course are shown above respective data points; arrows indicate time point of plasma exchange (PLEX/IA) for mAb-elimination. Black bar indicates mean iATP-values, red bar 3^rd^ percentile of healthy controls.

As depicted in figure 1, both German natalizumab-associated PML cases for whom blood samples were available at the time point of diagnosis exhibited very low intracellular ATP in CD4^+^-cells (iATP) in the range of the 3^rd^ percentile of healthy controls (235.3 ng/ml; PML 1 nataliz 243.5 ng/ml, PML 2 nataliz 238.9 ng/ml). Immediately after plasma exchange/immune adsorption (PLEX/IA) to eliminate remaining mAb, iATP increased strongly to normal values (fig. 1). This was clinically followed by development of IRIS 4 weeks later [9]. In the remaining 6 patients with natalizumab-associated PML, first samples were only available during/after PLEX (PML nataliz 3–8, fig. 1). In 4 of these patients, iATP was elevated during PLEX but decreased strongly after PLEX to low ranges (PML nataliz 3) or even below 3^rd^ percentile of HC (PML nataliz 4 and 5, fig. 1).

**Figure 2:**
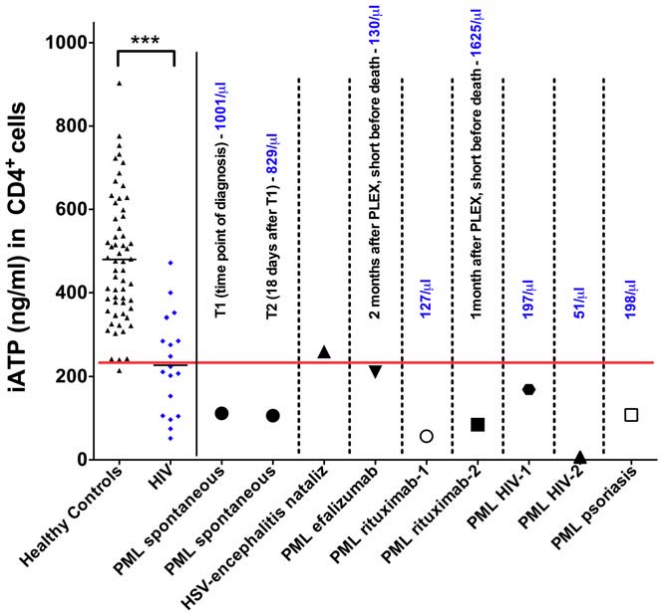
Intracellular CD4^+^-ATP-concentrations in mAb-associated opportunistic CNS-infections. Patients with progressive multifocal leukoencephalopathy (PML) without or with preceeding mAb-therapy. iATP levels of HIV patients were significantly lower than the HC cohort (p<0.0001). For patients with PML occurring under efalizumab or rituximab therapy and HSV-encephalitis under natalizumab, samples were only available after PLEX, as indicated. All other patients had not received PLEX. Total number of CD4^+^-cells per microliter (µl) and clinical course are shown above respective data points. HIV: HIV-patients at risk for opportunistic infections (see table 1 for details). Black bar indicates mean iATP values and red bar 3^rd^ percentile of healthy controls.

HIV patients at risk for opportunistic infections exhibited significantly lower iATP (227.2±27.7 ng/ml, p<0.0001) in comparison to HC (479.9±19.8 ng/ml). Likewise, strongly decreased iATP below/at 3^rd^ percentile of HC was also evident for a spontaneous case of PML (PML spontaneous 111.5 and 106.2 ng/ml, fig. 2), and the HIV-associated PML cases (PML HIV-1 168.9 ng/ml, PML HIV-2 6.6 ng/ml, fig. 2). Furthermore, a psoriasis patient with PML while undergoing treatment with fumaric acid (PML psoriasis 108 ng/ml, fig. 2) and a patient with natalizumab-associated herpes simplex virus-encephalitis (HSV-encephalitis nataliz 260.2 ng/ml, fig. 2) revealed dramatically decreased iATP-levels. Strongly reduced iATP was also apparent in a patient with rituximab-associated PML (PML rituximab-1 56.9 ng/ml, fig. 2) and two patients with lethal PML-outcome after rituximab- (PML rituximab-2 195.2 ng/ml, fig. 2) and efalizumab- (PML efalizumab 210.4 ng/ml) therapy respectively, tested several weeks after PLEX (fig. 2). In samples from all 16 patients with opportunistic CNS-infections before or at least one week after PLEX, iATP was reduced by 59% in comparison to HC (194.5±29 ng/ml, p<0.0001). Fourteen of these 16 patients had very low levels at/below 3^rd^ percentile of HC.

Each CD4^+^-iATP sample was also analyzed without PHA-stimulation with no major differences observed (data not shown). We addressed further the question of whether iATP as a functional measure of immune cellular responsiveness is independent of total CD4^+^ cell counts in the different cohorts analysed. For this purpose we correlated iATP vs. total CD4^+^ cell counts in n = 286 samples - apart from patients with opportunistic infections - consisting of healthy controls, MS patients devoid of immunomodulatory therapies and MS patients with natalizumab treatment. Value of iATP as a measure of responsiveness rather than depending on total cell numbers was reflected by the low correlation coefficient (R^2^ = 0.07). iATP in CD8^+^-cells analyzed in 10 patients with opportunistic CNS-infections did not demonstrate clear differences, nor did CD8^+^-cell numbers (CD8^+^-cells in HC 462.1±33.6/µl, in opportunistic CNS-infections 578.5±90/µl).

### CD4^+^-iATP reveals higher sensitivity than CD4^+^-cell counts in PML-patients, in patients with opportunistic CNS-infections and HIV-patients

**Figure 3:**
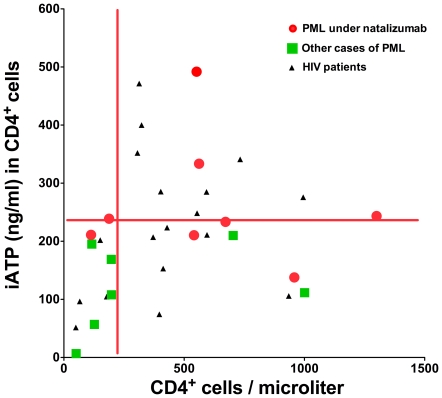
Relationship between total number of CD4^+^-cells and intracellular CD4^+^-ATP-concentrations. The X-axis depicts CD4^+^ -cell number per µl, y-axis intracellular CD4^+^-concentrations (iATP; ng/ml) of patients with PML and HIV-patients (black dots) at risk for opportunistic infections. Red dots indicate natalizumab-associated PML, green dots other PML cases (HIV-/rituximab-/efalizumab-associated or without previous immunotherapy). Red lines: 3^rd^ percentile of healthy controls.

Figure 3 correlates CD4^+^-iATP with CD4^+^-cell counts in patients with PML (mAb-associated and other etiologies) and the HIV-cohort. iATP was independent from CD4^+^-cell count (Pearson r = 0.19, R^2^ = 0.04, p = 0.3). Whereas a strong reduction of iATP was observed in all but two patients, only 7 of 15 patients with opportunistic CNS-infections, for whom cell counts were available exhibited reduced CD4^+^-cell numbers.

### Effects of plasma exchange (PLEX) on CD4^+^-iATP

To further investigate if the increase of iATP observed in natalizumab-associated PML-patients after plasma exchange was a direct effect of PLEX, we also analyzed patients undergoing PLEX for treatment of other neuroimmunological diseases. Five, of whom 4 were MS patients and one patient suffering from a specific from of peripheral neuropathy with monoclonal gammopathy, were analyzed for CD4+-iATP at the time-points described in the section containing patients' characterics.

According to the therapy consensus groups' recommendation in Germany, Switzerland and Austria (MSTKG), for MS patients not responding to primary relapse therapy with high dose intravenous corticosteroids, PLEX is an appropriate therapeutic escalation option. Monoclonal gammopathy of undetermined significance (MGUS) is a paraproteinemic disorder and a possible cause for slowly progressive symmetric distal sensorimotor neuropathy. PLEX is indicated as a therapeutic option for this neurologic disorder.

Also in these patients iATP was increased by 62% (714.4±122 ng/ml) when compared to concentrations prior to PLEX (441.5±103 ng/ml, p<0.04). This indicated that PLEX-associated increase of iATP in PML patients was at least partly independent from the removal of natalizumab and dynamics of JC virus infection and supports previous reports on direct effects of PLEX on parameters of cellular immunity [21]–[23].

### CD4^+^-iATP correlates with mitochondrial transmembrane potential (ΔΨ_m_) as functional measure of cellular energetics

To corroborate iATP-data with a biochemically independent assay, mitochondrial transmembrane potential (ΔΨ_m_) was investigated in CD4^+^-cells by flow-cytometry. A clear correlation of ΔΨ_m_ with iATP-levels could be demonstrated in the analysed samples (iATP/ΔΨ_m_−correlation:tau = 0.49, p = 0.03, n = 12). Tetramethyl rhodamine methylester (TMRM), which was used for this purpose, is particularly suitable to assess ΔΨ_m_ as its hydrophilic structure allows crossing plasma membranes. At the same time, its cationic character leads to accumulation in negatively charged compartments as it is the case for the inner mitochondrial membrane.

### Natalizumab may affect CD4^+^-iATP in a time dependent manner

**Figure 4:**
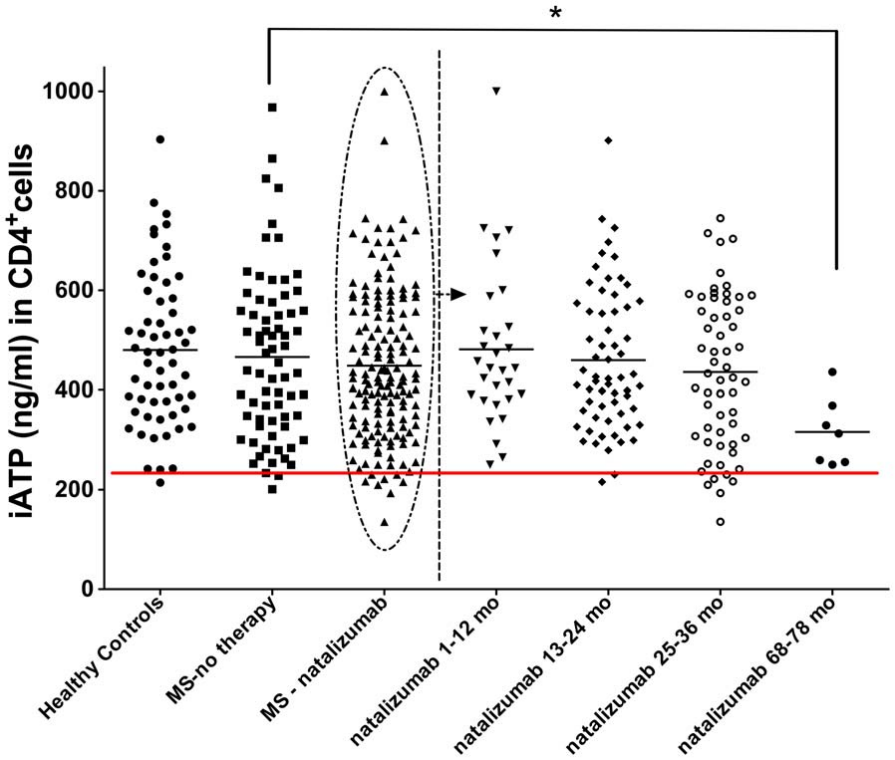
Intracellular CD4^+^-ATP-concentrations (iATP) of natalizumab-treated Multiple Sclerosis patients. Cross-sectional iATP in multiple sclerosis (MS) patients without immunotherapy (n = 70) or under natalizumab-treatment (n = 157). Right side of graph: analysis of natalizumab-treated MS-patients according to treatment duration given in months (mo). When compared to MS patients devoid of any immunomodulatory treatment, MS patients undergoing natalizumab therapy for more than 68 months revealed a moderately significant decrease of CD4^+^-iATP levels (*p = 0.04). Black bars: mean iATP values, red bar indicates 3^rd^ percentile of healthy controls.

As depicted in figure 4, in cross sectional natalizumab-treated patients (mean therapy duration 23 months, range 1–78) mean iATP revealed no significant difference (448.7±12 ng/ml) in comparison to healthy controls (HC, 479.9±19.8 ng/ml) or untreated MS-patients (465.9±19.8 ng/ml). Also *in vitro* experiments with natalizumab short term incubation vs. isotype control did not show any alteration in CD4^+^-iATP levels (data not shown). However, when separated according to therapy duration, a trend towards a decrease in mean iATP was observed in patients treated for more than 24 months (436±20.2 ng/ml at 25–36 months, patients treated for less than 24 months: 466.7±15.4 ng/ml, p = 0.2, fig. 4). 7 patients who had received natalizumab for more than 6 years already during the pivotal phase III-SENTINEL trial exhibited reduced mean iATP (316.2±26 ng/ml at 68–78 months, p = 0.04, fig. 4, right side). In addition, whereas 2/92 (2%) patients who were treated for less than 24 months revealed very low iATP at or below the 3^rd^ percentile of HC, 10/58 (17%) of the patients treated for more than 24 months had such low iATP-concentrations. Preliminary data on a small number of patients (n = 6) who discontinued natalizumab after 23.5 months of therapy (mean ± 2.8 months) indicates an increase of iATP after treatment cessation (iATP before treatment discontinuation 413±64.3 ng/ml, iATP after cessation 570.8±79.9 ng/ml). This observation supports our findings on decreased iATP dependent on natalizumab treatment duration, with potential reversal after treatment cessation.

## Discussion

Despite an increasing number of mAb-associated PML cases, so far no surrogate parameters have been identified that can effectively assist in individual disease risk assessment. Our main finding is strongly reduced iATP as a bioenergetic parameter of CD4^+^ T-cell function in mAb-associated PML-patients and patients with pathogenetically diverse opportunistic CNS-infections. CD4^+^ T-cell function, as determined by iATP proved to be more sensitive than mere CD4^+^-cell counts, a finding that was further supported by assessment of mitochondrial transmembrane potential as a biochemically independent method. Finally, our data indicates a decrease of iATP in MS-patients treated with natalizumab for more than 24 months, an interval during which an increased risk of PML development has been observed.

MAb-associated PML is likely the result of a complex combination of several pathogenic steps. Some commonly cited hypotheses involve alterations in peripheral cell-mediated immunity, mobilization of JCV-carrying CD34^+^-hematopoetic stem cells/pre-B-cells, neurotropic JCV-transformation, and mechanisms at the blood-brain barrier/CNS-parenchyma [35]–[39]. Here we have focussed on peripheral cellular immune functions as disorders that predispose to PML (e.g. HIV and leukemia) mainly involve defects of the peripheral immune system. In addition, several lines of evidence point to the important role of an efficient CD4^+^- and CD8^+^-cellular immune response in the control of JC-virus induced PML [11]–[14]. In HIV-associated PML, an antigen-specific CD8^+^-cell response in which the antigen was presented by an MHC class I molecule encoded by he HLA-A*0201 allele has been shown to correlate with prognosis and disease outcome [15], [16]. Various studies have concluded that specific anti-JC virus antibody responses are not sufficient to prevent or restrict PML [23], which further support the key role of cellular immunity that is needed in response to JCV infection.

Intracellular ATP serves as a measure of CD4^+^ T-cell functionality that correlates well with other markers of T-cell activation such as cytokine secretion and proliferation [18], [19]. The iATP level detected after mitogen stimulation, as performed in our assay, may represent the maximum excitability of the CD4^+^ effector cells; although not antigen-specific, the observed iATP level may resemble an activation level analogous to that occurring during an acute infection. Several lines of evidence indicate cellular hyporesponsiveness and not mere cell number as basis for our findings: (i) no difference in iATP between patient groups was observed in unstimulated CD4^+^-cells. (ii) there was no relationship between CD4^+^-iATP and CD4^+^-cell numbers. (iii) Finally, iATP-findings were also corroborated by analyses of mitochondrial transmembrane potential as a biochemically independent measure that allows a more ‘real time’ monitoring of mitochondrial function. Our data on higher sensitivity of iATP in comparison to CD4^+^-cell counts is well in line with initial reports of natalizumab-associated PML where no aberrant leukocyte/lymphocyte cell counts were evident [27]–[29]. Unaltered iATP in CD8^+^-cells and CD8^+^-cell counts in PML may reflect their physiologic role which comprises restraining the infection in the CNS *in situ*, but only secondarily after the initial immune response that occurs upon JCV antigen recognition by CD4^+^-cells and B-cells.

We also observed a strong influence of PLEX on iATP in natalizumab-associated PML. PLEX rapidly elimininates natalizumab and restores lymphocyte migratory function. However, the PLEX-associated increase of iATP appears to be at least partly independent from mAb removal, as indicated by the observed iATP increase in patients undergoing PLEX as a treatment for non-mAb-associated neuroimmunological diseases. Moreover, as these non-mAb-associated neuroimmunological diseases include conditions other than PML, the finding of CD4^+^-iATP increase upon PLEX strengthens the hypothesis that this phenomenon is specific to the PLEX treatment itself rather than due to changing dynamics of the JCV infection. This effect of PLEX on CD4^+^-iATP has not been previously shown, but is consistent with studies reporting leukocyte proliferation and induction of suppressor cell function upon PLEX treatment [21]–[23], [30], [31]. Both, direct PLEX effects on lymphocyte activation as well as the specific removal of natalizumab that restores the ability of immune effector cells to enter the JC-virus-infected CNS may be clinically relevant. Thus, PLEX was followed by development of IRIS 4 weeks later, arguing for effective resurrection of the immune system that can restrain JCV propagation in infected glial cells [9].

Although not classified as an immunosuppressive drug, a line of evidence demonstrates that natalizumab may actively inhibit immune effector cells: mAb-mediated blockade of the α_4_ integrin has been shown to lead to induction of apoptosis in murine thymocytes *in vitro* [32] and of T lymphocytes in an animal model of autoimmune-mediated neuritis *in vivo* [33]. This putative inhibition of immune effectors by natalizumab is unlikely to be mediated through an immediate effect on cellular bioenergetics, as we did not observe differences in iATP in our cross-sectional cohort of MS-patients or in our *in vitro* analyses. However, we did find a modest decrease in mean iATP in patients treated for more than 24 months, suggesting a treatment duration-dependent effect. This was particularly evident for 7 patients who had received natalizumab for more than 68 months, including treatment during the pivotal phase III-SENTINEL trial [18]. In fact, amongst those patients receiving natalizumab for more than 24 months, there was also a subset of individuals who actually had iATP levels that were below the 3^rd^ percentile of HC iATP. These findings could indicate that a small subgroup of patients may develop impaired T-cell function upon prolonged natalizumab treatment. Given the increased risk of PML development (1 in 600) with increased natalizumab treatment duration, that has been recently reported by the FDA [34], it is intriguing to speculate this increased risk may, at least in part, be related to an impairment of T-cell function arising with natalizumab administration over time. However, a larger patient cohort needs to be investigated longitudinally to corroborate our initial findings and to further assess the correlation between the increased risk of PML (that is associated with treatment duration) and the apparent decrease in iATP.

In conclusion, although the pathomechanisms of PML remain elusive, our data indicate that bioenergetic parameters relating to CD4^+^-mediated immunity may assist in neuroimmunological disease risk stratification and monitoring under mAb-immunotherapy, and may be a more sensitive measure than the CD4^+^ cell count, that is the only suggested measure of immunocompetence under natalizumab therapy to date [40]. Our data also indicate that iATP is a measure of immune cell responsiveness that is independent of total cell numbers, as consistently shown in our study and in the initial study performed to obtain FDA approval for the use of iATP measurements in the context of transplantation [41]. In order to determine the validity of CD4^+^-iATP in predicting PML a prospective study in which iATP levels are ascertained from pre-PML samples is required. However, given the current incidence of natalizumab-associated PML such a study would necessitate the involvement of thousands of individuals. It is conceivable, that bioenergetic measurements, particularly in conjunction with other parameters such as JC-viruria/viraemia, CD34^+^-cell numbers and molecular JCV-monitoring [4], could contribute to disease prediction. The assessment of anti-JCV antibodies with presumably high negative predictive value [26] with the longitudinal determination of iATP levels that potentially harbours a high positive predictive value, could constitute a clinically useful combination of assays.

## Methods

### Patients and sample collection

The study was approved by the ethics committee of the Ruhr-University Bochum and investigations were performed after written informed consent. All patients were devoid of acute infections (clinically, white blood cell count, C-reactive protein). Clinical characteristics of mAb-associated PML-cases (n = 11), two cases of HIV-associated PML, and one case each of PML without previous immunotherapy, a patient with psoriasis who developed PML under immunosuppressive therapies and fumaric acids and natalizumab-associated herpes-simplex encephalitis (HSV-E) are summarized in the tables 1–3. Two MS-patients who developed PML following natalizumab monotherapy (PML 1 and 3 nataliz, fig. 1) were recently reported [9], [10]. Clinical characteristics of respective control groups are given in table 1 (59 healthy individuals (HC), 70 therapy-naïve MS-patients). Of 157 natalizumab-treated MS-patients investigated, seven had on average 75 months (range 68–78 months) of natalizumab exposure since participation in the pivotal SENTINEL- phase III clinical trial [24]. 12 *de novo* natalizumab-treated MS-patients were followed longitudinally for up to 1 year. 5 patients undergoing PLEX as escalating therapy for severe relapse (4 glucocorticosteroid-refractory MS-relapses and one monoclonal gammopathy; 3–5 PLEX-cycles, 30 ml per kg body weight, 5% human albumin) were analyzed longitudinally. Eighteen HIV-patients were at risk for opportunistic infections despite highly active antiretroviral therapy (HAART) on clinical grounds due to previous infections, e.g. candidiasis, pneumonia and herpes simplex viral infections (according to the classification of Centers for Disease Control and Prevention (CDC) one patient with B2, 2 with B3 and 13 with C3, see table 1 for details).

**Table 1:** Characteristics/clinical data of subjects analyzed for iATP – Controls.

	Age, gender, [gender ratio (f∶m)], course of disease	Previous disease and therapy/therapy duration
**Healthy Controls (n = 59)**	36.7±11.5 [1.36]	NA
**MS – no therapy (n = 70)**	40.8±13.2 [1.8] 44 RRMS, 20 SPMS, 6 PPMS	NA
**MS – natalizumab (n = 157)**	37±9.5 [2.3] 157 RRMS	92 with up to 24 months, 58 with more than 24 months and 7 with 68–78 months of natalizumab-monotherapy
**HIV (n = 18)**	45.7±12.2 [0.2]	CDC classification: 1 with B2, 2 with B3, 13 with C3. 5 Patients had Kaposi's sacroma (HHV8 related), two oral hairy leucoplacia (EBV-related), 4 candida-esophagitis, 6 oral candidosis, 2 molluscs, 6 Pneumocystis jirovecii pneumonia, 3 chronic Herpes simplex infections, 1 cryptosporidosis, 1 toxoplasmosis

Individuals assessed for iATP measurement including healthy controls and MS Patients as respective control groups (multiple sclerosis (MS)-patients untreated, or natalizumab-treated). HIV patients (clinical presentation according to CDC classification) were tested as control patients at risk for opportunistic infections. NA = not applicable.

**Table 2 pone-0018506-t002:** Clinical data of subjects analyzed for iATP – Natalizumab associated PML.

	Age, gender	Previous disease and therapy/therapy duration	Clinical presentation of PML	Latency between first symptoms and diagnosis	Time point of ATP-measurement during course of PML	PML diagnosis, treatment, current status
**PML Natalizumab 1**	52, male	MS (diagnosed 1992), 1 year of intermittent interferon beta-treatment, 5 years of azathioprine (50 mg/d), 17×natalizumab	Initially impairment of cognition in terms of forgetfulness and inappropriate perception and answers when asked, mood alterations with aggressive episodes. After about 2 months of drowsiness, inattention, severe flaccid left-sided hemiparesis	PML diagnosed about two months after initial symptoms	Initial iATP measurement about two months after initial symptoms, before initiation of therapy, 2. iATP 5 days hereafter, 3. iATP four weeks after initial iATP, 4. iATP 4 months after first measurement, 5. iATP 4.5 months after first measurement	cMRI, CSF: JC-virus PCR (repeated). Plasma exchange and immunoadsorption, mirtazapine, mefloquine. Stable after IRIS, treated on an outpatient basis
**PML Natalizumab 2**	49, male	MS (diagnosed 2006), 5 years of intermittent interferon beta-treatment, 2 years of mitoxantrone therapy (cumulative dose of 85 mg/m^2^), 29×natalizumab	Paresthesia in the extremities, impairment of visual acuity on both eyes in terms of field defect due to retrochiasmatic lesion, dyslexia	PML diagnosed about two weeks after initial symptoms	Initial iATP measurement about two weeks after initial symptoms, before initiation of therapy, 2. iATP 12 days hereafter, 3. iATP 35 days after initial iATP	cMRI, CSF: JC-virus PCR (repeated). Plasma exchange and immunoadsorption, mirtazapine, mefloquine. Stable after IRIS
**PML Natalizumab 3**	37, male	MS (diagnosed 1999), 16×natalizumab	Myoclonic jerks of the left arm	PML diagnosed about two months after initial symptoms	Initial iATP measurement about two and a half weeks after initial symptoms, after plasma exchange and immunoadsorption, 2. iATP 12 days hereafter	cMRI, CSF: JC-virus PCR (repeated). Plasma exchange and immunoadsorption, mirtazapine, mefloquine. Stable after IRIS, treated on an outpatient basis
**PML Natalizumab 4**	57, male	MS (diagnosed 2006), 12×natalizumab	Initially: cognitive deficits and depression. During the course of PML drowsiness, right hemianopsia, aphasia, flaccid right-sided hemiparesis	PML diagnosed about 1 week after initial symptoms, initial ATP-measurement after 5×1000 methylprednisolone and 2 cycles of immunoadsorption	Initial iATP measurement about two and a half weeks after initial symptoms, before, 2. iATP 12 days hereafter	cMRI, CSF: JC-virus PCR (repeated). 5×1000 methylprednisolone and 4 cycles of immunoadsorption, mirtazapine, mefloquine, stable after IRIS
**PML Natalizumab 5**	42, female	3.5 years of intermittent interferon-treatment, 23×natalizumab	Epileptic seizures	PML diagnosed about 2 weeks after initial symptoms	iATP measurement about 6 weeks after first symptoms and 3 weeks after 5 cyles of plasma exchange	cMRI, CSF: JC-virus PCR. Plasma exchange, 5×1000 methylprednisolone, mefloquine, valproate, levetiracetam, stable after IRIS, treated on an outpatient basis
**PML Natalizumab 6**	32, male	MS (diagnosed 1997), 7 years of interferon beta-treatment, 34×natalizumab	Initially: depression, right sided hemiataxia and hemiparesis, gait ataxia. During the course of PML drowsiness and dysarhria	PML diagnosed about 2 weeks after initial symptoms	Initial iATP measurement about 4 weeks after first symptoms and 1.5 weeks after 6 cyles of plasma exchange, 2. iATP measurement 4.5 months hereafter	cMRI, CSF: JC-virus PCR. 6 cycles of plasma exchange, 5×1000 methylprednisolone, 3×30 g IVIG, mirtazapine, mefloquine, IRIS
**PML Natalizumab 7**	43, female	NK	NK	NK	Initial iATP measurement 1 day after 5 cyles of plasma exchange, 2. iATP-measurement 1 week after plasma exchange	NK
**PML Natalizumab 8**	35, female	MS (diagnosed 1993), 2 years of interferon beta-treatment, participation at SENTINEL trial, 5 cycles mitoxantrone (each 12 mg/m^2^ body surface), azathioprine (50 mg/d), 31×natalizumab	Impairment of visual acuity on both eyes, after immune reconstitution syndrome flaccid paresis of the left arm	PML diagnosed about 1 week after initial symptoms	iATP measurement 1.5 weeks after initial symptoms and 2 cycles of immunoadsorption	cMRI, CSF: JC-virus PCR. Immunoadsorption, 3×1000 methylprednisolone, mirtazapine, mefloquine, stable after IRIS, treated on an outpatient basis

MS Patients tested for iATP who developed PML under natalizumab immunotherapy. IRIS = immune reconstitution inflammatory syndrome, NA = not applicable, NK = not known.

**Table 3:** Clinical data of subjects analyzed for iATP – PML and HSVE with various underlying diseases and/or monocloncal antibodies.

	Age, gender	Previous disease and therapy/therapy duration	Clinical presentation of PML/HSVE	Latency between first symptoms and diagnosis	Time point of ATP-measurement during course of PML	PML/HSVE diagnosis, treatment, current status
**HSVE Natalizumab**	45, female	MS (diagnosed 1991), 6×natalizumab, 1997–1998 IFNβ-1a i.m., 1998–1999 mitoxantrone, 1999–2003 methotrexate	Global aphasia, impairment of cognition, drowsiness	HSVE diagnosed within two days after initial symptoms	iATP measurement within first week of initial symptoms and after initiation of aciclovir treatment	cMRI, CSF: HSV-virus PCR. 14 days of aciclovir, stable, treated on an outpatient basis
**PML spontaneous**	70, female	None known, no drug treatment	Dysphasia, memory disturbance, personality change, central facial palsy	PML diagnosed about two weeks after initial symptoms	Initial iATP-measurement about two weeks after first symptoms, 2. iATP 18 days hereafter	cMRI, CSF: JC-virus PCR. Mirtazapine, mefloquine, stable, treated on an outpatient basis
**PML efalizumab**	47, male	Psoriasis, fumaric acids, 3 years of methotrexate, 3 years of efalizumab	Progressive flaccid left-sided hemiparesis and hemihypesthesia	PML diagnosed about two weeks after initial symptoms	iATP measurement 1 month after 5 cycles of plasma exchange, shortly before death	cMRI, CSF: JC-virus PCR. 5 cycles of plasma exchange, 3×80 g IVIG, mirtazapine, mefloquine, IRIS, death
**PML rituximab 1**	60, male	Mantle cell lymphoma (diagnosed November 2008), 3 cycles of R-CHOP therapy (including rituximab), 3 cycles of R-DHAP therapy	Progressive global aphasia and right-sided hemiparesis	PML diagnosed about three weeks after initial symptoms by histopathological analysis of CNS tissue after brain biopsy	iATP measurement about 9 months after initial symptoms	cMRI, CSF: negative JC-virus PCR, brain biopsy. Dexamethasone, cidofovir
**PML rituximab 2**	67, male	Follicular lymphoma CD20^+^ (diagnosed 2000), adjuvant radiochemotherapy, rituximab	Brachiofacial left-sided hemiparesis followed by impairment of cognition, mood alterations with aggressive episodes three months later	PML diagnosed about four months afterinitial symptoms	iATP measurement about 9 months after initial symptoms, shortly before death	cMRI, CSF: JC-virus PCR. Mirtazapine, mefloquine, valganciclovir, duloxetin, death
**PML HIV 1**	38, male	HIV (diagnosed 1994), CDC classification C3, HAART until February 2009, at time point of PML diagnosis: HIV RNA in plasma 544 copies/µl, HIV RNA in CSF 328 copies/µl	Psychosis, impairment of cognition, mood alterations, followed by right-sided hemiparesis, aphasia and epileptic seizures	PML diagnosed about four weeks after initial symptoms	iATP measurement about 8 weeks after initial symptoms, before initiation of antiretroviral therapy	cMRI, CSF: JC-virus PCR. Mirtazapine, mefloquine, lopinavir, ritonavir, epivir, retrovir
**PML HIV 2**	38, male	HIV (diagnosed December 2009, at the time point of PML diagnosis), HIV RNA in plasma 790 copies/µl,	Motor aphasia, progressive right-sided hemiparesis, internuclear ophtalmoplegia (INO) to the left side	PML diagnosed about six weeks after initial symptoms	iATP measurement about 4 weeks after diagnosis	cMRT, CSF: JC-virus PCR, brain biopsy. Cidofovir, mirtazapine, initiation of HAART
**PML psoriasis immuno-suppressed**	57, female	Psoriasis (diagnosed about 30 years ago), pulmonary sarcoid stage II (diagnosed 2005), oral corticosteroids (2005–2006), methotrexate (2006) and fumaric acids	Gait and truncal (left-sided) ataxia, fatigue	PML diagnosed few days after onset of ataxia	iATP measurement 2 weeks after initial symptoms	cMRI, CSF: JC-virus PCR, brain biopsy, cidofovir, mirtazapine

Clinical characteristics and disease course of patients with or withour preceeding immunodeficient conditions and/or mAb-immunotherapy who developed PML or Herpes simplex virus encephalitis (HSVE) tested for iATP.

### Assessment of ATP production in CD4^+^ and CD8^+^ cells

After stimulation of sodium-heparin collected whole blood samples (phytohaemagglutinin, PHA, 2.5 µg/ml, 15 h; Sigma-Aldrich, Taufkirchen, Germany) CD4^+^-cells were immunoselected using magnetic beads (Invitrogen, Karlsruhe, Germany). Following cell lysis, ATP-concentrations were measured in a FDA-approved luciferase-activity based assay according to the manufacturer's specifications in quadruplicates (ImmunoKnow™, Cylex, Columbia, USA). Between day coefficient of variation (CV) was 9.3% in HC including natural biological variation over several months. ATP-concentration in CD8^+^-cells was assessed following the same protocol in an off-label experimental assay. For *in vitro* experiments, whole blood samples of healthy controls and MS-patients (n = 9) were incubated for 24 hours with natalizumab (BiogenIdec, Cambridge, USA) with a concentration of 5 µg/ml, equivalent to serum levels under the therapeutic dose of 300 mg natalizumab-infusion in MS-patients. As isotype control antibody (isoAb) IgG4 (Sigma, Deisenhofen, Germany) was used.

Absolute cell numbers (CD4^+^/CD8^+^/CD19^+^/CD56^+^) were determined by flow cytometry (FACS-Calibur, Becton Dickinson, Heidelberg, Germany). Trucount™ tubes containing Trucount™ control beads (Becton Dickinson) were used as internal standard for absolute cell count determination in the lymphocyte gate. Due to logistical reasons cell counts could not be obtained in the patient with natalizumab-associated HSV-encephalitis.

### Flow cytometric analysis of mitochondrial transmembrane potential (ΔΨ_m_) in CD4^+^ cells

Changes of mitochondrial transmembrane potential (ΔΨ_m_) in PMA-treated (2.5 µg/ml, 30 min; Sigma-Aldrich) CD4^+^-cells (anti-CD4-APC, Becton Dickinson) of HC and HIV (n = 12) were measured by flow cytometry of mitochondrial tetramethyl rhodamine methylester load (TMRM - 2 µM, 10 min; Invitrogen) [25]. Mean fluorescence intensity (MFI) of TMRM in CD4^+^-labelled cells was assessed with a minimum of 10.000 CD4^+^-cells analyzed (Canto II, Becton Dickinson).

### Statistical analysis

Two-sided parametric t-test was performed for all statistical analyses except Pearson correlation test for iATP/CD4^+^-cell numbers (GraphPad Prism, La Jolla, USA) and Kendall's Tau for iATP/ΔΨ_m_-correlation (SPSS). p<0.05 (*), p<.001 (**) and p<.0001 (***) were considered significant. Data is given as mean ± standard error (SEM).
